# Numerical Study to Evaluate the Flexural Performance of Concrete Beams Tensile Reinforced with Fe-Based Shape Memory Alloy Rebar According to Heating Temperature

**DOI:** 10.3390/ma18081703

**Published:** 2025-04-09

**Authors:** Ki-Nam Hong, Sang-Won Ji, Yeong-Mo Yeon

**Affiliations:** Department of Civil Engineering, Chungbuk National University, Chungbuk 28644, Republic of Korea

**Keywords:** Fe-based shape memory alloy (Fe-SMA) rebar, flexural behavior, heating temperature, recovery stress, finite element (FE) analysis

## Abstract

An Fe-based shape memory alloy (Fe-SMA) is an alloy that has a characteristic of being able to return to its original shape when heated, even after undergoing plastic deformation. Many researchers have conducted various studies to understand the effectiveness of using Fe-SMA in concrete structures. Most studies selected the heating temperature of Fe-SMA to be below 160 °C based on the logic that concrete hydrolyzes when its temperature exceeds 160 °C. However, because the recovery stress of Fe-SMA increases as the heating temperature increases, it is expected that greater prestress could be introduced when the heating temperature is high. In this study, to confirm this, a numerical study was conducted to evaluate the effect of Fe-SMA heating temperature on the flexural performance of concrete members through finite element (FE) analysis. The analysis results showed that the initial crack load of the specimen increased by about 89% to 173% as the heating temperature of Fe-SMA increased. In addition, the accuracy of the proposed FE model (FEM) was verified through experiments. As a result, it was confirmed that the proposed FE analysis can relatively accurately predict the failure mode and load–displacement relationship of the specimen.

## 1. Introduction

To tackle the challenges associated with reinforced concrete (RC), such as rebar corrosion triggered by cracking, the use of prestressed concrete (PSC) has been adopted. PSC applies a pre-compressive force to the structure through tendons, which can be strands, wire, steel bars, or braids. A structure that incorporates a pre-compressive force proves to be effective in controlling cracks. This is because the compressive force is introduced into the tensile zone of the concrete, and deflection can be efficiently managed because of the upward displacement resulting from the pre-compressive force [1,2,3]. However, there is a drawback in that the tensile force applied to the structure decreases over time due to concrete drying shrinkage, relaxation, and creep. In particular, structures that use prestressing methods such as pre-tensioning and bonded post-tensioning cannot be re-tensioned [4,5]. Moreover, even for structures that use unbonded post-tensioning systems, re-tensioning may be difficult due to the complexity and safety concerns involved in the process.

In order to address this limitation of PSC, numerous researchers have explored the possibility of introducing prestress into structures using shape memory alloys (SMAs) [6,7,8,9,10]. SMA is a special alloy that possesses the ability to revert to its original shape, a phenomenon known as the shape memory effect (SME) [11]. This occurs through activation, which involves heating or cooling, even in the face of plastic deformation that exceeds the elastic range under an external load [12]. If an SMA that has been pre-tensioned is activated after its deformation has been restrained, it is unable to revert to its original shape. Instead, a compressive force, known as recovery stress, is generated within the alloy due to the SME [13,14]. Based on this principle, if a pre-tensioned SMA is embedded within the concrete and is subsequently activated, a compressive force is introduced into the structure. This is because the recovery stress is generated by the bonding force between the concrete and the SMA.

Generally, SMAs based on nickel–titanium (Ni-Ti), commonly referred to as nitinol, have primarily been used in various industries such as the military, aerospace, and medical fields [15,16,17,18]. However, nitinol presents several challenges as a construction material, including high raw material costs, complex manufacturing processes, and a low-temperature history [19,20]. Conversely, Fe-SMA is a more economically viable option than nitinol, primarily because it is Fe-based and its manufacturing process is simple [21,22]. Consequently, numerous studies have been undertaken to explore the potential of incorporating Fe-SMA as a construction material.

Shahverdi et al. [23] conducted an experimental study in which they used Fe-SMA strips as reinforcement by applying the near-surface mounted (NSM) method. They prepared specimens of concrete beams reinforced with Fe-SMA strips using the NSM method and assessed their flexural performance through a four-point loading test. The analysis of the results from this research indicated that the specimen reinforced with Fe-SMA using the NSM method exhibited a higher initial stiffness and initial crack load compared with the specimen reinforced with carbon fiber reinforced polymer (CFRP) using the NSM method, as well as the non-reinforced specimen. This was explained as being due to the introduction of a prestress force into the specimen through the recovery stress of Fe-SMA. In a separate study, Yeon et al. [24] conducted an experimental investigation using Fe-SMA bars as reinforcement in a new structure. They conducted a four-point loading test with one-way slabs that were tensile reinforced with Fe-SMA bars. The experimental variables they considered included the activation of Fe-SMA, the amount of Fe-SMA reinforcement, and the reactivation of Fe-SMA. They reported that the initial load causing a crack in the specimen with activated Fe-SMA was higher than that in the specimen with non-activated Fe-SMA. However, Fe-SMA activation did not significantly affect the ultimate load. Additionally, they subjected one-way slabs, which were tensile reinforced with Fe-SMA, to up to 70% of the ultimate load in order to reactivate the Fe-SMA. The research suggested that the deflection and cracks resulting from external loads could be somewhat restored through the reactivation of Fe-SMA. An experimental analysis by Choi et al. [25] was conducted to assess the effect of prestressing Fe-SMA wires that were embedded in mortar beams. Mortar specimens reinforced with Fe-SMA wires were prepared with dimensions of 30 mm width, 20 mm thickness, and 200 mm length. Their behavior was assessed using a three-point loading test. It was found that the initial stiffness and initial crack load of the specimen with activated Fe-SMA wires increased by 35% and 45%, respectively, in comparison to the specimen with non-activated Fe-SMA. Shahverdi et al. [26] conducted a study on the material properties of Fe-SMA. They prepared Fe-SMA strips of 100 and 50 mm in width and 1.5 and 0.5 mm in thickness and performed tensile and recovery stress tests on them. They found that the Fe-SMA strips exhibited a high recovery stress as the heating temperature increased. However, the rate of increase in recovery stress started to decline once the activation temperature surpassed 300 °C.

Previous studies involving the use of Fe-SMA as a construction material can be categorized into the following: (1) studies that assessed the material properties of Fe-SMA as a construction material [27,28,29]; (2) studies that used Fe-SMA plates, bars, and strips as reinforcement for the NSM and external bonding methods [19,30,31,32,33,34]; (3) studies that employed Fe-SMA rebars as tensile reinforcement in new structures [35,36]; and (4) studies that induced confined effects in concrete columns using Fe-SMA [37,38]. Among these, certain studies introduced a prestress force through the bond between Fe-SMA and the cement matrix. This includes studies that employed Fe-SMA for the NSM method and those that used Fe-SMA as reinforcement for new structures. These studies conducted experiments with the heating temperature of Fe-SMA capped at 160 °C or lower. This was performed to avoid thermal damage to the Fe-SMA reinforcement-concrete interface at high temperatures [35,39,40]. Many studies investigating the material properties of Fe-SMA have reported that the recovery stress increases as the activation heating temperature rises [41,42]. Therefore, an increase in the heating temperature for Fe-SMA activation would allow the introduction of a greater prestressing force into concrete beams reinforced with Fe-SMA, based on the aforementioned principle. However, no studies have been conducted to experimentally determine the impact of the heating temperature of Fe-SMA on the flexural behavior of concrete beams reinforced with Fe-SMA rebar. Accordingly, this study evaluates the effect of the activation heating temperature of Fe-SMA bars on the flexural performance of concrete beams reinforced with Fe-SMA. To achieve this, direct tensile tests and recovery stress evaluations were conducted to experimentally assess the recovery stress of Fe-SMA under different heating temperatures. Based on the results of the tensile tests and recovery stress measurements, a stress–strain model for Fe-SMA was proposed. Using this model, an FEM was developed to evaluate the flexural behavior of RC beams reinforced with Fe-SMA bars. The FEM was then used to analyze the influence of the heating temperature on the flexural performance of the beams. Finally, beam specimens with the same specifications as those used in the numerical analysis were fabricated, and flexural tests were performed to verify the validity of the proposed FEM.

## 2. Finite Element (FE) Analysis Model

### 2.1. FE Model (FEM)

As outlined in Table 1, heating or non-heating and heating temperature (150, 200, 250, and 300 °C) were considered as experimental variables in this study. In Table 1, the specimen name ‘SM’ signifies specimens reinforced with Fe-SMA rebars. ‘RT’ refers to a specimen with unheated Fe-SMA. The number after the letter ‘T’ represents the heating temperature for Fe-SMA.

**Figure 1:**
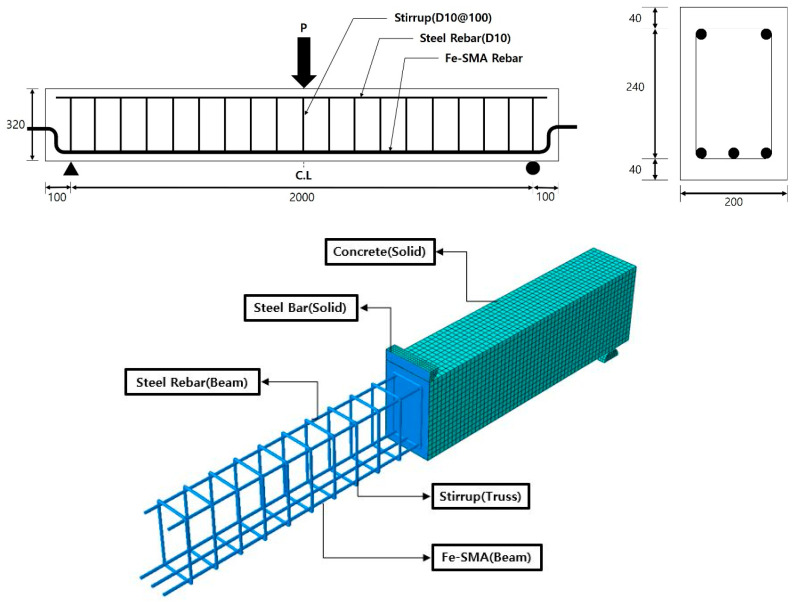
Test specimen.

In order to confirm the flexural behavior of concrete beams tensile reinforced with Fe-SMA, based on the heating temperature of Fe-SMA, an FE analysis was performed using ABAQUS/CAE 6.13-6 [43], a comprehensive FE analysis software. The proposed FE analysis model is shown in Figure 1. The specimen had dimensions of 200 mm in width, 320 mm in height, and an effective depth of 265 mm, as depicted in Figure 1. The total length of the specimen was 2200 mm, and its net span was 2000 mm, because of the placement of supports 100 mm from both ends. To prevent shear fracture from occurring before the specimens were fractured by bending, U-shaped stirrups made from SD400 grade D10 deformed bars were positioned at intervals of 100 mm.

The load and support bars were modeled as 8-node solid elements, with an element size of 20 mm. The stirrup was modeled as a 2-node truss element with an element size of 20 mm, while the compressive reinforcement and Fe-SMA rebar were modeled as 2-node beam elements also with an element size of 20 mm. It was assumed that the Fe-SMA rebars, compressive reinforcement, and stirrups were fully bonded to the concrete. To achieve this, the ‘Embedded region’ and ‘General contact’ commands were used, and ‘Normal Behavior’ was applied to prevent the load and support bars from infiltrating into the concrete solid element.

The loading process was executed through displacement control, which computed the internal force by applying vertical displacement to the load bar. During the displacement control analysis, the increment of displacement was set to 0.005 mm for each step. As for the target of the analysis, loading was performed until the displacement of the load bar reached 35 mm.

### 2.2. Material Modeling

**Figure 2:**
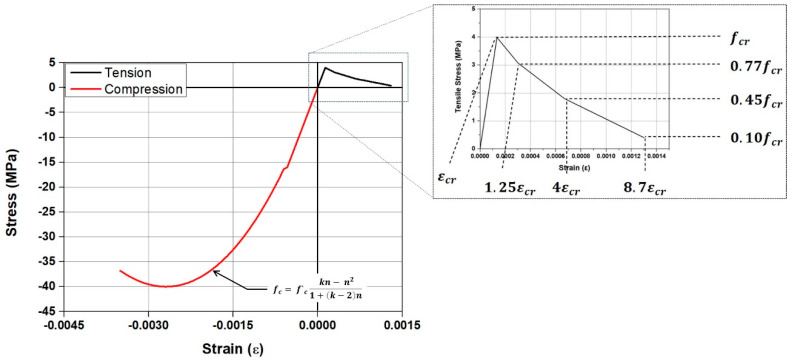
Stress–strain model of concrete.

Figure 2 illustrates the stress–strain relationship of the concrete material model used in this study. The Concrete Damaged Plasticity (CDP) model provided by ABAQUS/CAE 6.13-6 was employed as the concrete material model, and default values were input for the plasticity factors, as detailed in Table 2 [44]. The model recommended in Eurocode 2 was used to simulate the compressive behavior of the concrete in the CDP model, as presented in Equations (1)–(3). For the tensile behavior of concrete, the tensile hardening model, which is optimized for ABAQUS/CAE 6.13-6 and proposed by Wahalathantri [45], was employed. The tensile strength ($f_{cr}$) and Young’s modulus of concrete ($E_{c}$) were calculated using Equations (4) and (5) presented by the ACI code. In this study, the compressive strength of concrete (${f^{\prime }}_{c}$) was assumed to be 40 MPa.

(1) $$f_{c}={f^{\prime }}_{c}\frac{kn-n^{2}}{1+\left(k-2\right)n}$$

(2) $$k=1.15E_{c}\frac{\varepsilon_{c1}}{{f^{\prime }}_{c}}$$

(3) $$n=\frac{\varepsilon_{c}}{\varepsilon_{c1}}$$

(4) $$f_{cr}=0.63\sqrt{{f^{\prime }}_{c}}$$

(5) $$E_{c}=4700\sqrt{{f^{\prime }}_{c}}$$

where $f_{c}$ represents the stress of the concrete. $\varepsilon_{c}$ and $\varepsilon_{c1}$ denote the strain of the concrete and the strain at the point when the concrete reaches its compressive strength, respectively.

**Figure 3:**
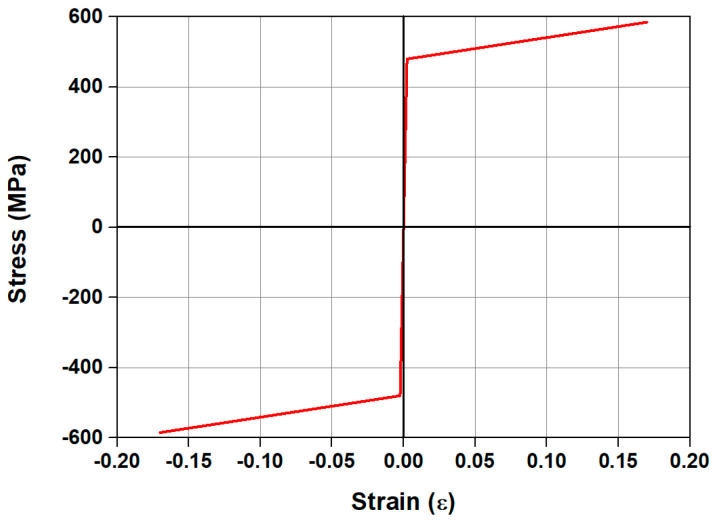
Stress–strain model of the steel rebar.

Figure 3 presents the material model of the steel rebar that was used for the compressive reinforcement and stirrups. A bilinear model was used, which demonstrates the elastic behavior before the yielding of the steel rebar and the elasto-plastic behavior after the yielding of the steel rebar. In this study, a steel rebar material model was established assuming that SD 400 grade rebars were used.

**Figure 4:**
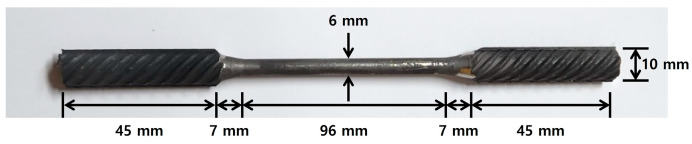
Fe-SMA rebar test specimen for the direct tensile test.

**Figure 5:**
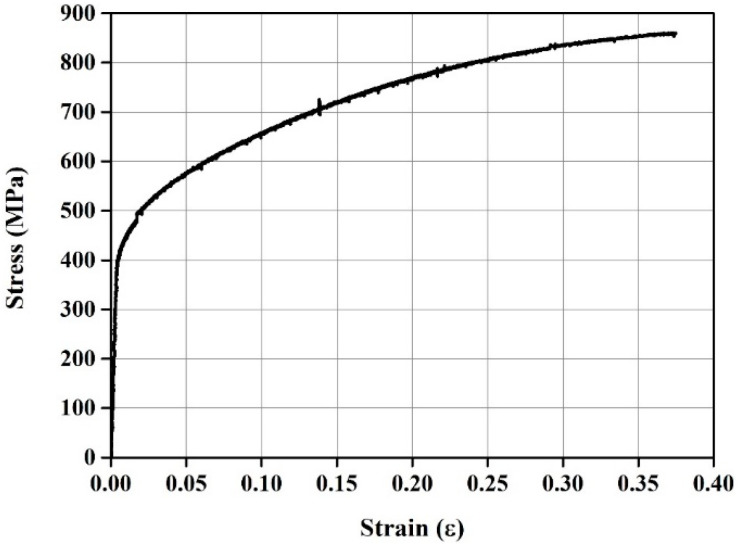
Stress–strain curve of the Fe-SMA rebar.

The Fe-SMA rebar used in this study, manufactured by Company E in Switzerland, has a chemical composition of Fe–17Mn–5Si–10Cr–4Ni–1(V, C) (wt%) [46]. To assess mechanical properties of the Fe-SMA rebar, the rebar was fabricated into a dog bone-shaped specimen. The specimen has a central diameter of 6 mm, with a gauge length of 96 mm and a grip section of 45 mm as shown in Figure 4. A direct tensile test was conducted on this specimen. During the test, the displacement was controlled at a rate of 0.5 mm/min using a 100 kN universal testing machine (UTM). The strain of the specimen was measured using a strain gauge, and the data were collected every second through a data acquisition system (DAQ). Figure 5 presents the stress–strain relationship of Fe-SMA derived from the direct tensile test. The Young’s modulus, ultimate strength, and elongation of the Fe-SMA specimen were 123.7 GPa, 856.16 MPa, and 38%, respectively.

**Figure 6:**
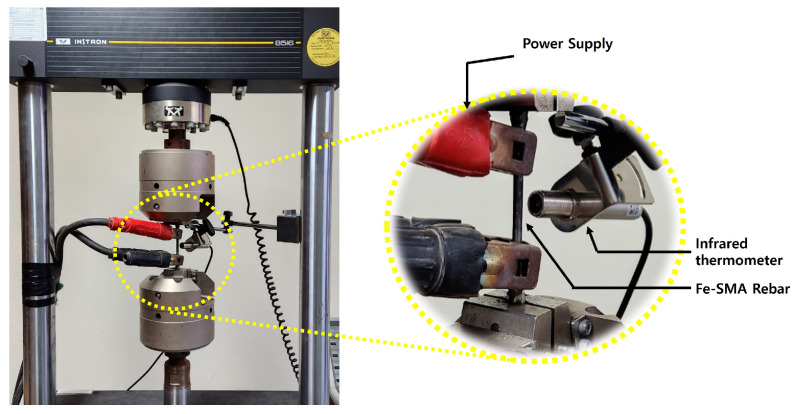
Overview for evaluating the recovery stress of Fe-SMA rebar.

The recovery stress of the Fe-SMA rebar was assessed using a specimen identical to that used in the direct tensile test. According to Hong et al. [42], the prestrain of Fe-SMA has no significance on recovery stress, and Lee et al. [47] reported that the corrosion resistance of Fe-SMA significantly decreases if the prestrain of Fe-SMA exceeds 4%. Consequently, in this study, the prestrain of Fe-SMA rebar was assumed to be 4%. The test specimen was pre-tensioned to 4% at a displacement control rate of 0.2 mm/min using a 100 kN UTM. To prevent buckling due to initial thermal expansion, a prestress of approximately 50 MPa was applied to the test specimen. Following this, the displacement of the machine was kept constant. The Fe-SMA specimen was then subjected to heating until it reached the target temperatures (150, 200, 250, and 300 °C). This was achieved using electrical resistance with a current supply of 2 A/mm^2^. Once the surface temperature of the specimen reached the target, the power supply was interrupted, allowing the specimen to cool to room temperature. During the heating and cooling process, the temperature of the test specimen was measured using a non-contact thermal sensor. Once the specimen had cooled to room temperature, it was stretched until it fractured with the displacement controlled at a rate of 0.2 mm/min. The data measured during the test were collected every second using DAQ. Figure 6 shows the test overview for evaluating the recovery stress of Fe-SMA rebar.

**Figure 7:**
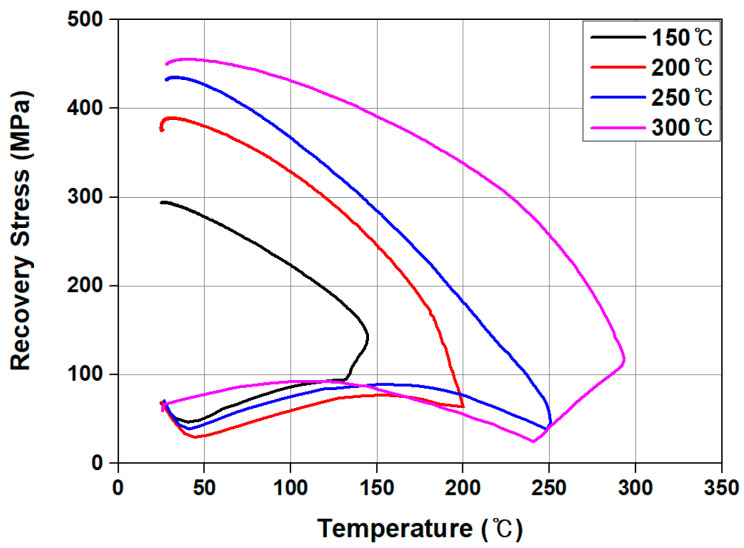
Temperature–recovery-stress curves according to the heating temperature.

Figure 7 illustrates the relationship between temperature and recovery stress for the Fe-SMA specimen measured during heating to the target temperature and subsequent cooling to room temperature. The figure indicates that the stress in the test specimen slightly decreased due to thermal expansion when the temperature reached approximately 35 °C at the start of heating. As heating continued, recovery stress was generated. This stress slightly decreased because of thermal expansion once the temperature of the test specimen exceeded approximately 160 °C. When the power was turned off after the test specimen reached the target temperature, the specimen cooled down. During this cooling process, recovery stress was generated through the SME and thermal contraction until the test specimen reached room temperature. Finally, the Fe-SMA test specimen heated to 150 °C, 200 °C, 250 °C, and 300 °C exhibited recovery stress values of 296 MPa, 389 MPa, 435 MPa, and 455 MPa, respectively, indicating that the recovery stress increased with increasing temperature.

**Figure 8:**
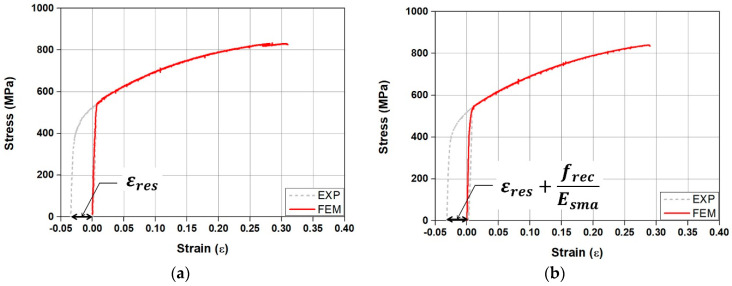
Stress–strain model of the Fe-SMA: (**a**) non-Activation; (**b**) activation.

The material model of Fe-SMA was established using data from the stress–strain relationship obtained through the tensile test. In this case, for the stress–strain relationship of the non-activated Fe-SMA, pre-strain was initially generated, followed by offsetting the residual strain ($\varepsilon_{res}$) to zero, as depicted in Figure 8a. For the stress–strain relationship of the activated Fe-SMA, the *x*-axis was offset by taking into account the residual strain, recovery stress ($f_{rec}$), and Young’s modulus of Fe-SMA ($E_{sma}$), as illustrated in Figure 8b. The activation of Fe-SMA was modeled using temperature and the thermal expansion coefficient in the predefined field, allowing the Fe-SMA rebar to contract as the temperature increased. Hosseini et al. [48] reported that the recovery stress of Fe-SMA decreased compared to that of Fe-SMA under ideal restraint conditions when thermal expansion of the matrix occurred. Additionally, Yeon et al. [24] and Hong et al. [35] reported that when Fe-SMA is activated inside concrete, the recovery stress applied to concrete is 78% to 82% of the theoretical value. According to these preliminary findings, the Fe-SMA material modeling process, the thermal expansion coefficient was adjusted so that the recovery stress of the Fe-SMA matched 80% of the actual recovery stress shown in Figure 7.

## 3. FE Analysis Results

### 3.1. Failure Mode Predicted by FEM

**Figure 9:**
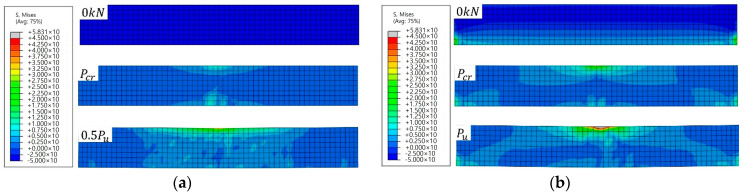
Stress distribution of the specimens. (**a**) SM–RT; (**b**) SM–T300.

Figure 9 illustrates the distribution of stress in SM-RT and SM-T300 prior to loading, at the time of cracking ($P_{cr}$), and at the ultimate load ($P_{u}$). In this context, the initial cracking load was determined as the point at which the concrete stress exceeded the tensile strength calculated using Equation (4), while the ultimate load was defined as the point at which the applied load on the specimen reached its maximum value. As indicated in Figure 9b, compressive stress was observed at the bottom of SM-T300 because of the recovery stress of Fe-SMA before loading. This recovery stress led to the SM-T300 exhibiting initial cracking under a higher load. Consequently, a higher stress was generated in the loading zone than in SM-RT. At the moment of initial cracking and ultimate load, a lower stress was generated in the region where the Fe-SMA rebar was situated for SM-T300 compared with SM-RT.

**Figure 10:**
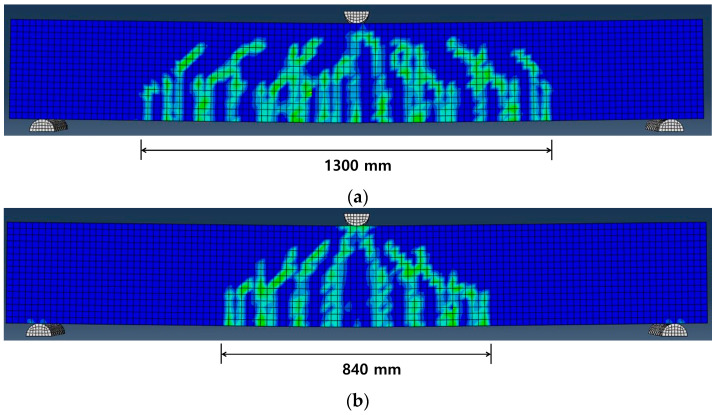

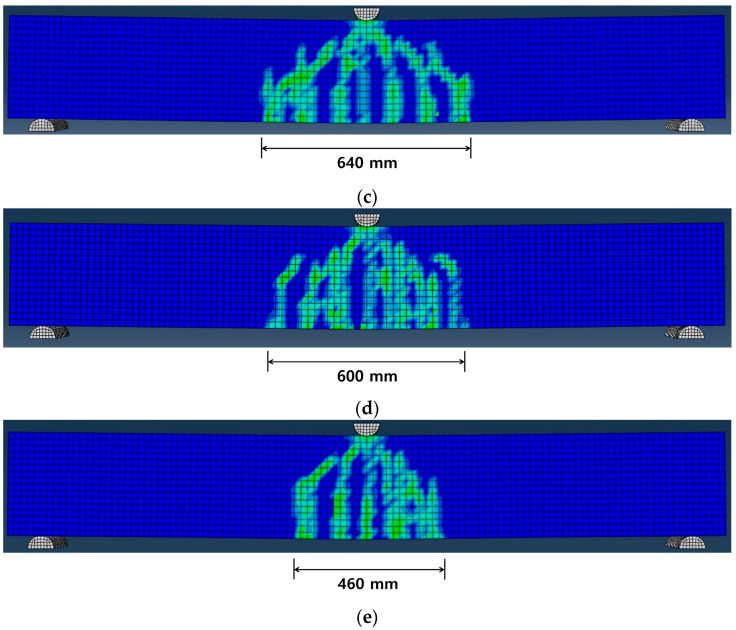
Crack pattern of the specimens predicted by FE analysis. (**a**) SM-RT; (**b**) SM-T150; (**c**) SM-T200; (**d**) SM-T250; (**e**) SM-T300.

Figure 10 presents the tensile damage pattern of concrete, derived using DAMAGET, to investigate the crack pattern of the specimen. As shown in Figure 10a, SM-RT exhibited a typical flexural failure pattern, in which the initial cracks formed in the tensile region gradually propagated toward the compressive zone. In Figure 10, the crack pattern of the proposed FE analysis model anticipated a reduced number of cracks in the specimens with an increase in the heating temperature. In addition, the crack distribution width of the specimens with heated Fe-SMA rebars was reduced to 35.4–64.6% compared to the SM-RT specimens, and it became narrower as the heating temperature increased. The reduction in the number of flexural cracks appears to be due to the introduction of prestress forces into the specimens, as the recovery stresses generated by the activation of Fe-SMA acted as compressive forces.

### 3.2. Load-Carrying Capacity Predicted by FEM

**Figure 11:**
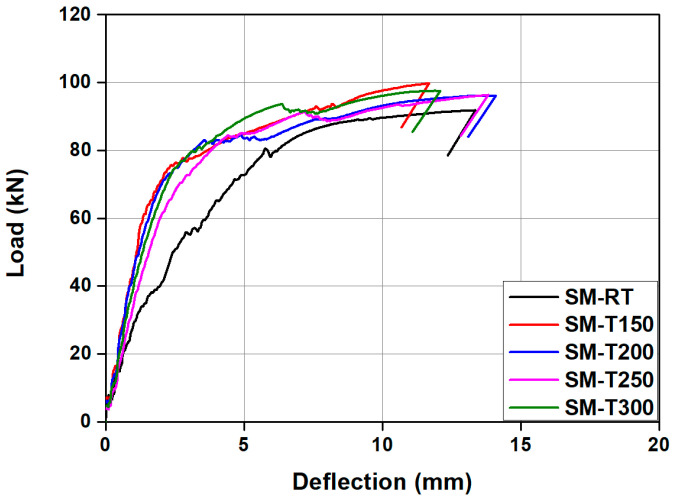
Comparison of load–displacement relationships of test specimens according to Fe-SMA heating temperature predicted by FE analysis.

**Figure 12:**
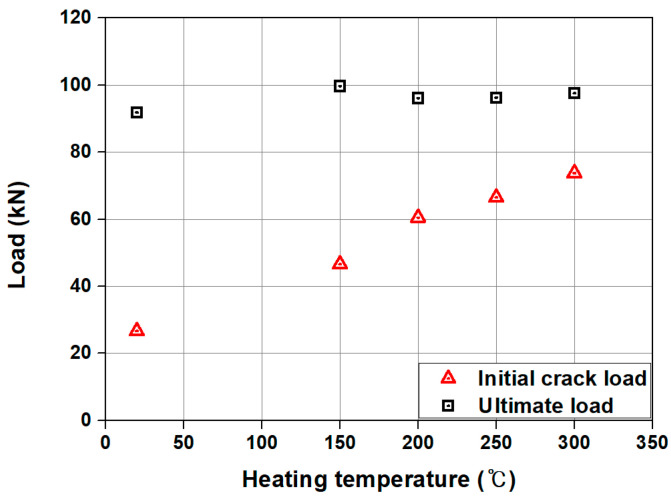
Comparison between loading stages for heating temperature of Fe-SMA rebar.

Table 3 and Figure 11 show a summary of the analysis results and comparison of the load–displacement relationship of the specimens predicted by FE analysis, respectively. In the case of the SM-RT, initial cracking was observed at approximately 26.63 kN. As indicated in Figure 11, SM-RT did not display a clear yield point. This is likely due to the fact that, unlike conventional steel materials, as shown in Figure 5, Fe-SMA does not have an obvious yield point. The ultimate load for the SM-RT was found to be 91.83 kN. As shown in Figure 12, the initial crack loads for the SM-T150, SM-T200, SM-T250, and SM-T300 were 46.57, 60.47, 66.49, and 73.76 kN, respectively, representing an increase of 74.9% to 177.0% over that of SM-RT. The increase in the initial crack load appears to be due to the recovery stress generated by the activation of the Fe-SMA rebar, which served as a compressive force for the specimens. Conversely, the ultimate loads of the specimens varied minimally, only between 4.7% and 8.6%, irrespective of whether they were heated or not and regardless of the heating temperature. This indicates that the intensity of the applied compressive force is more effective in enhancing the usability of the beams, rather than improving their load-carrying capacity, as is the case with conventional PSC members.

## 4. Experimental Program for FEM Validation

### 4.1. Test Specimens

To verify the accuracy of the proposed FEM, the flexural behavior of a concrete beam using Fe-SMA as a tensile reinforcement was evaluated. Five specimens were prepared. The geometry of the specimen is the same as that used in the FE analysis shown in Figure 1. As shown in Figure 1, the specimens were tensile reinforced with three 4% pre-strained Fe-SMA rebars. After all preliminary tasks were completed, the U-shaped stirrups and Fe-SMA rebars were assembled and insulated with insulation tape to prevent energy loss during electrical resistance heating. Subsequently, ready-mixed concrete was poured into the formwork to fabricate the specimens. The formwork was removed after 7 days, and the specimens were subjected to wet curing for 28 days.

### 4.2. Materials

The concrete used for creating the specimen had a design compressive strength of 40 MPa, and its mixing design properties are outlined in Table 4. During the concrete pouring process, test samples were prepared to measure the concrete’s compressive strength. These included five cylindrical samples with dimensions of Φ100 mm × 200 mm. These samples were demolded and cured under the same conditions as the main specimens. On the day of the bending test, concrete compressive strength tests were conducted according to American Standard for Test and Material (ASTM) C39/39M [49]. Analysis of the test results revealed that the average compressive strengths of the concrete were 40.6.

As depicted in Figure 1, the rebars used as compressive rebars and shear stirrups in the specimens were of the SD400 grade D10 variety. The Young’s modulus, yield strength, and an elongation of the steel rebars, as supplied by the vendor, are 200 GPa, 480 MPa, and 17.4%, respectively.

### 4.3. Test Setup

**Figure 13:**
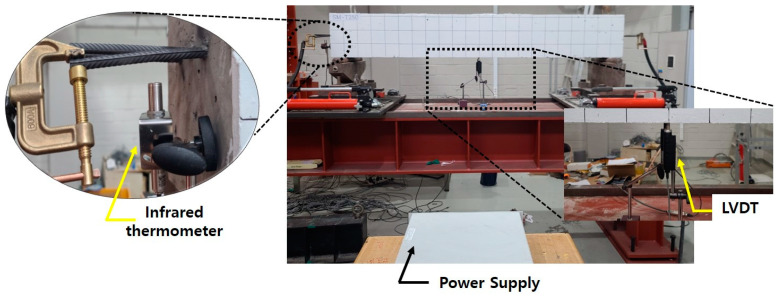
Overview of the activation system.

Figure 13 illustrates the heating setup of the Fe-SMA rebar. Before heating the Fe-SMA rebar, the specimen was positioned to achieve a net span of 2000 mm. The Fe-SMA rebar embedded within the specimen was activated via electrical resistance heating using a current of 5 A/mm^2^ using a DC power supply. The current was introduced by connecting copper electrodes to both ends of the Fe-SMA rebar, as shown in Figure 13. A non-contact K-type thermal sensor was used to monitor the heating process by measuring the temperature of the Fe-SMA rebar at the exposed section outside the specimen. Heating continued until the target activation temperature was reached, at which point the power supply was immediately turned off. The camber produced during the activation process was measured using a centrally installed linear variable differential transformer (LVDT) with a capacity of 10 mm, located at the bottom of the specimen. The data measured by both the thermal sensor and the LVDT were collected and stored every second using DAQ.

**Figure 14:**
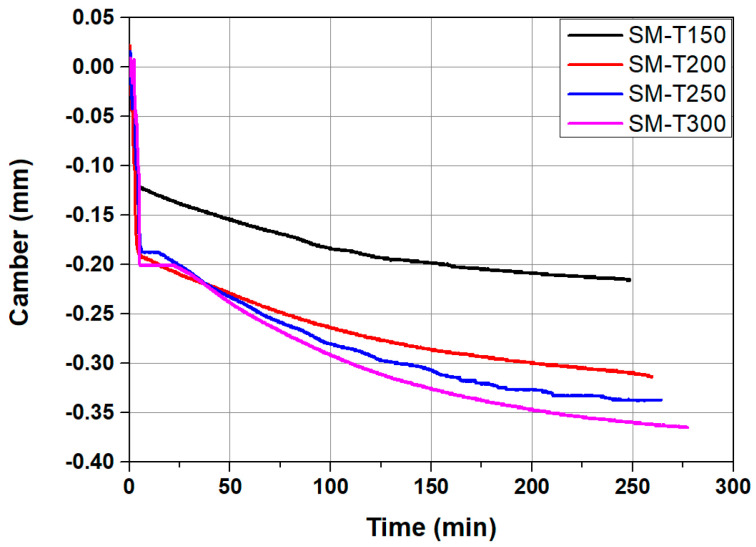
Time–displacement relationship according to heating temperature.

Figure 14 presents the time–displacement curves at the mid-span of the specimens during the activation of the Fe-SMA. As shown in Figure 14, a slight deflection (downward displacement) occurred immediately after the heating of the Fe-SMA rebar. This was due to thermal expansion, which typically occurs in steel materials during the initial heating stage, as illustrated in Figure 7. However, as the heating continued, camber (upward displacement) began to develop in the specimen. This behavior can be attributed to the shape memory effect (SME) induced by the temperature increase in the Fe-SMA. The SME was converted into recovery stress through the bond between the Fe-SMA and concrete, generating an eccentric compressive force on the concrete section. The final camber of the specimen SM-T150, which was activated by heating the Fe-SMA to 150 °C, was approximately 0.216 mm. In the case of SM-T200, in which the Fe-SMA was activated at 200 °C, the final camber increased to 0.365 mm, representing an approximate 69% increase compared to SM-T150.

**Figure 15:**
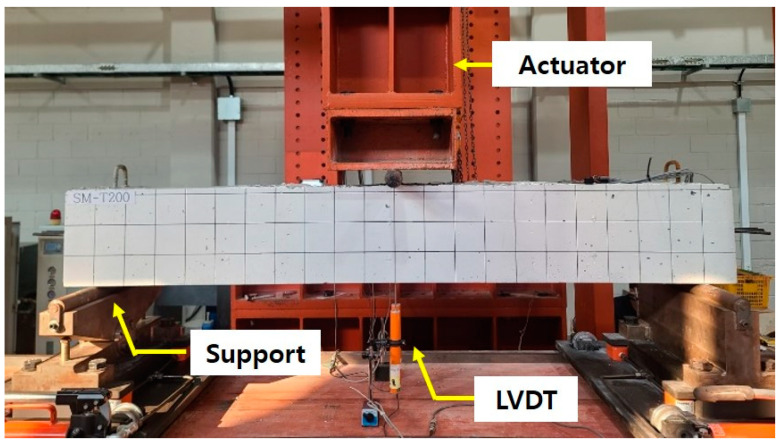
Three-point bending test setup.

To assess the flexural performance of the concrete beam after the cessation of upward deformation caused by the activation of the Fe-SMA rebar, a three-point bending test was conducted. During this test, a load was applied at a rate of 3 mm/min using the displacement control method. The resulting deflection of the specimen from loading was measured using two LVDTs, each with a capacity of 100 mm, centrally installed at the bottom of the specimen. Additionally, the initial cracks and their propagation during loading were visually monitored and recorded on the surface of the specimen. Figure 15 provides a visual representation of the setup for the three-point bending test.

## 5. Comparison of Experimental and Numerical Results

### 5.1. Failure Mode

**Figure 16:**
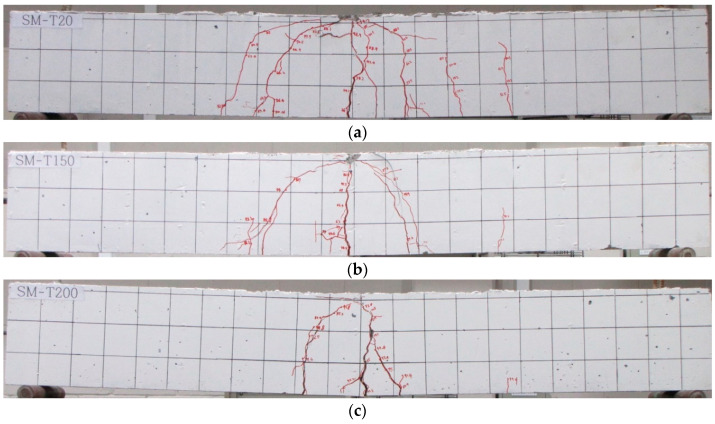

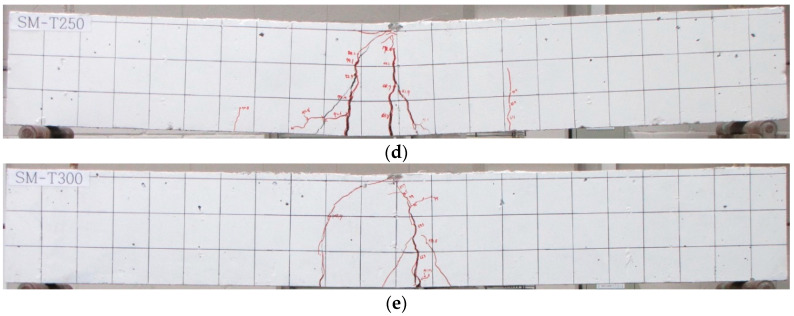
Failure mode of the specimens after experiments. (**a**) SM-RT; (**b**) SM-T150; (**c**) SM-T200; (**d**) SM-T250; (**e**) SM-T300.

Figure 16 illustrates the failure modes of the specimens at the end of the experiment. In the case of SM-RT, flexural cracks began to appear at the center of the bottom from the initial loading phase. As the test progressed, additional flexural cracks developed in the specimen. The specimen ultimately exhibited a typical flexural failure mode, characterized by the gradual propagation of initial flexural cracks, leading to the fracture of concrete in the compression zone. Specimens with activated Fe-SMA rebar also exhibited the typical flexural failure mode, where initial flexural cracks progressively propagate, resulting in a concrete fracture in the compression zone. In addition, consistent with the FE analysis results, a decrease in the number of cracks was observed in the specimens with activated Fe-SMA, with the cracks being more concentrated in the center, as compared to SM-RT, as shown in 14. Therefore, the proposed FE analysis model can relatively accurately predict the failure mode of the specimen according to the Fe-SMA heating temperature.

### 5.2. Load-Carrying Capacity

**Figure 17:**
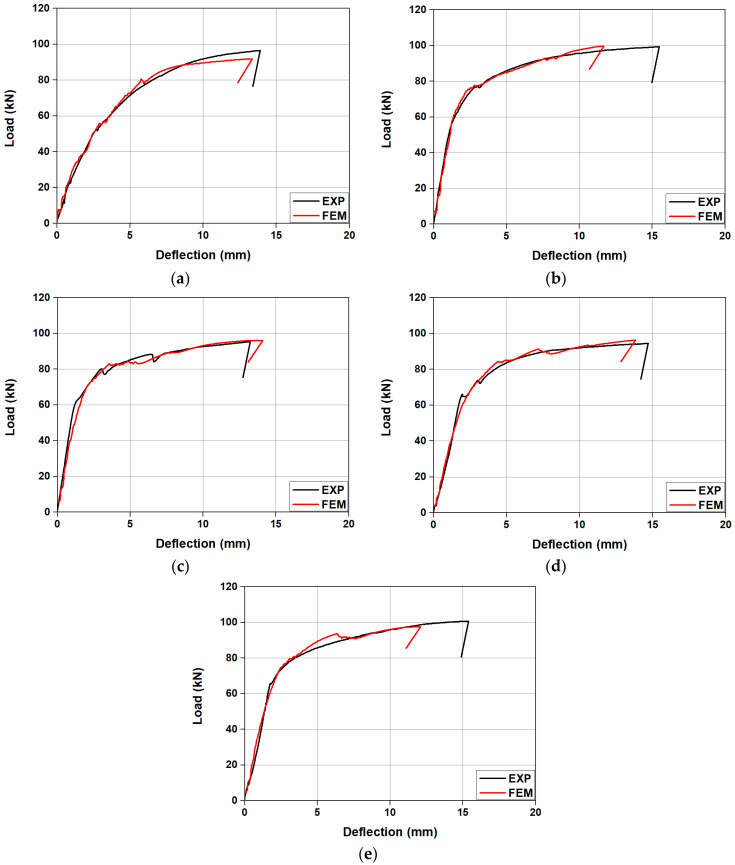
Comparison of load–deflection curves between experiment and FE model. (**a**) SM-RT; (**b**) SM-T150; (**c**) SM-T200; (**d**) SM-T250; (**e**) SM-T300.

Figure 17 provides a comparison between the load–deflection relationships of the specimens as predicted through FE analysis and the results from the experiment. A summary of these experiment results is presented in Table 5. The initial cracking loads presented in Table 5 were determined as the smaller value between the load at which the first visible crack was observed and the load at which a change in the initial stiffness occurred. In Figure 17, the load–deflection relationship of the specimens confirmed by the experiment is relatively close to the results predicted through FE analysis. For example, the initial crack load for SM-RT confirmed from the experiment was approximately 24.1 kN. This value showed an error of approximately 2.6 kN (10%) compared with FE analysis results. The ultimate load for SM-RT, confirmed by the experiment, showed a minor error of approximately 5% when compared with the value obtained from FE analysis results. The initial crack loads for SM-T150, SM-T200, SM-T250, and SM-T300 were 45.66, 59.56, 64.00, 65.74 kN. This indicates that the initial crack load increased with the rise in heating temperature, which can be attributed to the increase in recovery stress, mirroring the FE analysis results. The initial crack loads of the specimens obtained from the experiment showed an average error of just 6.05% when compared with the FE analysis results. Furthermore, the ultimate loads of SM-T150, SM-T200, SM-T250, and SM-T300, obtained from the experiment, were 99.3, 95.4, 94.44, and 100.68 kN, respectively. These results prove that the increase in Fe-SMA heating temperature does not have a significant effect on the ultimate load, similar to the result predicted by FE analysis. Also, the ultimate loads of the specimens, confirmed by the experiment, demonstrated an average error of merely 2.21% compared with the FE analysis results. Consequently, it is anticipated that the proposed FE analysis model will be able to predict the flexural behavior of concrete beams tensile reinforced with Fe-SMA with relative accuracy. This includes aspects such as stiffness, initial crack load, and ultimate load. This good agreement between the FEM predictions and the experimental results is mainly attributed to the accurate modeling of material properties and boundary conditions. In particular, the recovery stress of Fe-SMA was calibrated to 80% of the measured values to simulate the actual restraint conditions inside concrete, which significantly improved the reliability of the FEM analysis. However, in this study, bent Fe-SMA rebars, as shown in Figure 1, were used to prevent slip between the Fe-SMA rebar and the concrete. Accordingly, the FEM proposed in this study assumes perfect bonding between the concrete and the Fe-SMA rebar. In future research, potential damage at the interface between the concrete and the Fe-SMA rebar caused by increased heating temperatures, as well as the resulting degradation in bond strength, should be further considered. In addition, the activation temperature of the Fe-SMA rebar was limited to 300 °C, and only a concrete compressive strength of 40 MPa was considered in this study. Therefore, future research should thoroughly investigate the effects of thermal damage at the concrete interface caused by various temperatures, as well as the degree of thermal damage resulting from changes in concrete compressive strength, on RC beams reinforced with Fe-SMA rebars. By incorporating the resulting changes in bond characteristics and thermal damage effects into the material model and boundary conditions, more accurate and realistic structural analyses can be achieved.

## 6. Conclusions

This study conducted numerical research to assess the influence of heating temperature for Fe-SMA activation on the flexural behavior of concrete beams that were tensile reinforced with Fe-SMA rebars. The following conclusions were drawn from this study:

1. As a result of experimentally evaluating the recovery stress of Fe-SMA according to the heating temperature, the recovery stress of Fe-SMA heated to 150 °C, ~300 °C was 296 MPa~455 MPa, and the recovery stress increased as the heating temperature increased.
2. As a result of FE analysis, as the heating temperature of Fe-SMA increased, the number of cracks generated in the specimen decreased and the cracks were concentrated in the center of the specimen. This decrease in the number of cracks is believed to be the result of Fe-SMA developing a higher recovery stress when the heating temperature increased, introducing a greater prestress force into the specimen.
3. The initial crack load of the specimen with activated Fe-SMA showed an increase ranging from 89.7% to 173.2% in comparison to that of the specimen with non-activated Fe-SMA, and this increase was observed to be proportional to the heating temperature. Furthermore, in the final failure stage, both the number of cracks and the width of crack distribution decreased as the heating temperature of Fe-SMA increased.
4. Experimental results to verify the accuracy of the proposed FE analysis model; a decrease in the number of cracks and centralization of cracks were observed as the Fe-SMA heating temperature increased, as predicted in FE analysis.
5. The initial crack loads observed in the experiments showed a small discrepancy from the values predicted by the FEM, ranging from a minimum of 2% to a maximum of 11%, with an average error of 6.05%. Similarly, the ultimate loads predicted by the FEM exhibited only a minor difference from the experimental results, ranging from 0.4% to 5%, with an average error of 2.2%. Moreover, the load–deflection responses from the FE analysis were found to be in good agreement with the experimental results. Therefore, it is anticipated that the proposed FE analysis model will be capable of accurately predicting the flexural behavior of concrete beams that are tensile reinforced with Fe-SMA rebars.

## Figures and Tables

**Table 1:** Test variables.

Specimen	Tensile Reinforcement	Reinforce Ratio (%)	Heating Temperature (°C)
SM-RT	Fe-SMA rebar	0.482	-
SM-T150			150
SM-T200			200
SM-T250			250
SM-T300			300

**Table 2:** Concrete plasticity parameters [44].

Dilation Angle	Eccentricity	fb0/fc0	K	Viscosity Parameter
40	0.1	1.16	0.6667	0

**Table 3:** Summary of the FEM results.

Specimen	Initial Crack Load		Ultimate Load		Load Increase Compared to SM-RT (%)	
	Load (kN)	Deflection (mm)	Load (kN)	Deflection (mm)	Initial Crack (%)	Ultimate (%)
SM-RT	26.63	0.93	91.83	13.36	-	-
SM-T150	46.57	1.05	99.71	11.76	74.9	8.58
SM-T200	60.47	1.56	96.13	14.20	127.1	4.68
SM-T250	66.49	2.41	96.31	13.94	149.7	4.88
SM-T300	73.76	2.41	97.56	11.89	177.0	6.24

**Table 4:** Mixing properties of the concrete.

Slump (cm)	Air Content (%)	W/B (%)	S/a (%)	Weight Per Unit Volume (kg/m^3^)				
				W	C	S	G	AD
15	3.5	31.5	43.5	163	361	716	954	4.38

W/B—water–binder ratio; S/a—fine aggregate ratio; W—water; C—cement; S—sand; G—gravel; AD—admixture.

**Table 5:** Comparison of FEM and experiment results.

Specimen	Initial Crack Load (kN)		EXP/FEM	Ultimate Load (kN)		EXP/FEM
	EXP	FEM		EXP	FEM	
SM-T20	24.06	26.63	0.9	96.5	91.83	1.05
SM-T150	45.66	46.57	0.98	99.3	99.71	1.00
SM-T200	59.56	60.47	0.98	95.4	96.13	0.99
SM-T250	64	66.49	0.96	94.44	96.31	0.98
SM-T300	65.74	73.76	0.89	100.68	97.56	1.03
Average			0.94			1.01

## Data Availability

The raw data supporting the conclusions of this article will be made available by the authors on request.

## Abbreviations

The following abbreviations are used in this manuscript:

Fe-SMA	Fe-based shape memory alloy
FE	finite element
FEM	finite element model
NSM	near-surface mounted
CFRP	carbon fiber reinforced polymer
CDP	Concrete Damaged Plasticity
UTM	universal testing machine
DAQ	data acquisition system
